# Machine learning-based prediction of mortality in acute myocardial infarction with cardiogenic shock

**DOI:** 10.3389/fcvm.2024.1402503

**Published:** 2024-10-14

**Authors:** Qitian Zhang, Lizhen Xu, Zhiyi Xie, Weibin He, Xiaohong Huang

**Affiliations:** ^1^ Department of Cardiology, Zhangzhou Affiliated Hospital of Fujian Medical University, Zhangzhou, Fujian, China; ^2^ Department of Endocrinology, Shengli Clinical Medical College of Fujian Medical University, Fujian Provincial Hospital, Fuzhou University Affiliated Provincial Hospital, Fuzhou, China

**Keywords:** MIMIC-IV, eICU-CRD, acute myocardial infarction, cardiogenic shock, machine learning, hospital mortality

## Abstract

**Background:**

In the ICU, patients with acute myocardial infarction and cardiogenic shock (AMI-CS) often face high mortality rates, making timely and precise mortality risk prediction crucial for clinical decision-making. Despite existing models, machine learning algorithms hold the potential for improved predictive accuracy.

**Methods:**

In this study, a predictive model was developed using the MIMIC-IV database, with external validation performed on the eICU-CRD database. We included ICU patients diagnosed with AMI-CS. Feature selection was conducted using the Boruta algorithm, followed by the construction and comparison of four machine learning models: Logistic Regression (LR), eXtreme Gradient Boosting (XGBoost), Adaptive Boosting (AdaBoost), and Gaussian Naive Bayes (GNB). Model performance was evaluated based on metrics such as AUC (Area Under the Curve), accuracy, sensitivity, specificity, and so on. The SHAP method was employed to visualize and interpret the importance of model features. Finally, we constructed an online prediction model and conducted external validation in the eICU-CRD database.

**Results:**

In this study, a total of 570 and 391 patients with AMI-CS were included from the MIMIC-IV and eICU-CRD databases, respectively. Among all machine learning algorithms evaluated, LR exhibited the best performance with a validation set AUC of 0.841(XGBoost: 0.835, AdaBoost: 0.839, GNB: 0.826). The model incorporated five variables: prothrombin time, blood urea nitrogen, age, beta-blockers and Angiotensin-Converting Enzyme Inhibitors or Angiotensin II Receptor Blockers. SHAP plots are employed to visualize the importance of model features and to interpret the results. An online prediction tool was developed, externally validated with the eICU-CRD database, achieving an AUC of 0.755.

**Conclusion:**

Employing the LR algorithm, we developed a predictive model for assessing the mortality risk among AMI-CS patients in the ICU setting. Through model predictions, this facilitates early detection of high-risk individuals, ensures judicious allocation of healthcare resources.

## 1 Introduction

Acute myocardial infarction (AMI) with cardiogenic shock (CS) presents as a critical syndrome marked by a rapid decline in cardiac pump function, leading to systemic circulatory failure and multi-organ dysfunction (1). Despite a downward trend in the incidence and mortality of AMI (2, 3), patients presenting with concurrent CS continue to have a dire prognosis (4). In patients with AMI, the incidence of CS is approximately 5%–10% (5–7). This often leads to multi-organ dysfunction, including acute kidney injury, respiratory failure, and neurological complications (8). The 30-day mortality rate for AMI-CS approaches 40%, with the one-year mortality reaching up to 50%, indicating its exceedingly high lethality (7, 9, 10). AMI-CS patients frequently require emergency coronary interventions and mechanical support, increasing treatment complexity and cost (7). Accurately predicting mortality risk in AMI-CS patients is vital for guiding clinical decisions, enabling personalized treatment, and optimizing resources (11).

Currently, predictive models for in-hospital mortality among AMI-CS patients are primarily constructed using logistic regression methods (12–14). Commonly used severity scoring systems such as APACHE II, APACHE III, SAPS II, and SOFA often exhibit limited predictive performance (13, 14). The IABP-SHOCK II score and CardShock score model exhibit satisfactory performance in predicting mortality among CS patients, yet there are certain limitations regarding the size of the model construction cohort and external validation (15, 16). With the rapid progress of precision medicine, machine learning is increasingly utilized in healthcare for outcome prediction, diagnosis, medical image interpretation, and treatment (17–19). Machine learning exhibits superior clinical prediction accuracy and performance compared to traditional statistical methods, with the added advantage of faster processing speeds (20). With the advent of interpretable techniques like SHAP, users can gain a better understanding of the predictive outcomes generated by machine learning models (21). Currently, interpretable machine learning (ML) models for predicting in-hospital mortality in patients with AMI-CS have not been established.

Our study aims to utilize diverse machine learning algorithms to construct predictive models for assessing in-hospital mortality risk among AMI-CS patients. We identified machine learning models with superior predictive performance and clinical relevance, established an online predictive system, and conducted external validation. Furthermore, we employed the SHAP methodology to identify key clinical predictive factors and interpret the model outcomes.

## 2 Materials and methods

### 2.1 Data sources

This study utilized patient data from two databases: the Medical Information Mart for Intensive Care IV (MIMIC-IV) database and the eICU Collaborative Research Database dataset (eICU-CRD). MIMIC-IV (version 2.2) is an extensive critical care database, encompassing detailed records of over 190,000 ICU patients from 2008 to 2019. This dataset aggregates a wealth of clinical information, including patients’ demographic profiles, laboratory test results, medication histories, and additional comprehensive data sets (22). The eICU Collaborative Research Database (eICU-CRD) pools detailed data from more than 200,000 patients in various U.S. intensive care units, collected between 2014 and 2015, making it an essential tool for advancing research in critical care medicine (23).

### 2.2 Study population

This study focuses on patients with AMI-CS, and obtains the relevant patient data from two databases using ICD-9 and ICD-10 codes. The exclusion criteria for the study population are as follows: (1) under the age of 18, and (2) no ICU experience or an ICU stay less than 24 h. For patients with multiple admissions or a history of ICU stays, only the first ICU experience during their first admission is included. In our research, cardiogenic shock (CS) was defined based on several clinical parameters, primarily including the following: ① Systolic blood pressure (SBP) < 90 mmHg or the need for vasopressor support to maintain SBP ≥ 90 mmHg; ② Signs of reduced cardiac output leading to poor organ perfusion (e.g., low urine output, cold and clammy skin, altered mental status); ③ Exclusion of other causes of shock, such as hypovolemic or septic shock.

### 2.3 Data extraction and preprocessing

In this study, we included ICU patients diagnosed with AMI-CS, from whom we extracted the following data: (1) Demographics: age, gender, height, and weight; (2) Vital Signs: temperature (T), heart rate (HR), respiratory rate (R), systolic blood pressure (SBP), diastolic blood pressure (DBP), mean blood pressure (MBP), and peripheral oxygen saturation (SpO2); (3) Laboratory Indicators: complete blood count, liver and kidney function tests, electrolytes, lipid profile, blood gases, coagulation profile, and cardiac enzymes; (4) Comorbidities: hypertension, diabetes mellitus, hyperlipidemia, chronic obstructive pulmonary disease (COPD), pneumonia, chronic kidney disease (CKD), and atrial fibrillation (AF); (5) Surgical Indicators: coronary angiography (CAG), percutaneous coronary intervention (PCI), percutaneous transluminal coronary angioplasty (PTCA), and intra-aortic balloon pump (IABP); (6) Medication data: angiotensin-converting enzyme inhibitors/angiotensin II receptor blockers (aceiorarb), beta Blockers, furosemide, spironolactone, dobutamine, dopamine, epinephrine, milrinone, norepinephrine, and phenylephrine; and (7) Other Indicators such as Mechanical Ventilation and APACHE II score. The primary outcome was 28-day all-cause mortality, defined as death from any cause within 28 days starting from admission to the ICU.

The raw data is refined through advanced calculations. This involves computing Body Mass Index (BMI) from height and weight, and assessing in-hospital mortality within 28 days using hospitalization duration and survival status. In both the MIMIC-IV and eICU-CRD datasets, we rigorously addressed missing data by eliminating entries with missing rates exceeding 30%. Subsequently, we employed the K-Nearest Neighbors (KNN) imputation method to handle remaining missing values. KNN imputation leverages sample similarity, utilizing observed values from the K nearest samples to predict and fill missing values effectively. Exploration of variable relationships was conducted using Spearman correlation coefficients, visually represented through heatmaps. Multicollinearity among variables was meticulously assessed using Variance Inflation Factor (VIF) values. To optimize predictive performance and prevent overfitting, we selectively pre-screened predictive variables with high correlations or VIF exceeding 5.

### 2.4 Model construction and evaluation

After preprocessing, we initially included 38 predictive factors to construct a model for predicting in-hospital mortality within 28 days for patients with AMI-CS. To improve the stability of the predictive model, all continuous variables were standardized, scaled to have a mean distribution of 0 and a standard deviation of 1. The Boruta algorithm was used for feature selection. Its principle is to determine the most relevant features in the dataset based on the Random Forest by comparing with randomly generated “shadow” features. This method has the advantage of not requiring assumptions, which enhances the robustness of the model and simplifies the feature selection process (24). The XGBoost method was employed to rank the selected features based on their importance in a professional manner. The MIMIC dataset was used to construct the model, employing a 10-fold cross-validation method to generate training and validation sets. Four machine learning models were established and validated, including Logistic Regression (LR), eXtreme Gradient Boosting (XGBoost), Adaptive Boosting (AdaBoost), and Gaussian Naive Bayes (GNB). Model comparisons are conducted on the validation set. The Area Under the Receiver Operating Characteristic (ROC) curve (AUC) is utilized to assess the model's discriminative ability, while calibration curves and Brier scores are employed to evaluate model accuracy. Additionally, Decision Curve Analysis (DCA) curves are utilized to assess clinical utility. Additionally, confusion matrix metrics were included for evaluation, such as accuracy, sensitivity, specificity, positive predictive value, negative predictive value, and F1 score. We also compared the predictive performance of our model with commonly used severity scoring systems such as SOFA, APSIII, SAPSII, and OASIS.

### 2.5 Model interpretation

The SHAP (SHapley Additive exPlanations) method is an approach for explaining machine learning model predictions (25). It leverages Shapley values to decompose the impact of each feature on the model output, providing insights into the prediction process. By considering interactions between features, SHAP offers intuitive explanations for individual predictions, aiding in the identification of key features and prediction sources. In SHAP plots, red and blue points represent the SHAP values of each sample, with red indicating higher feature values and blue indicating lower feature values. Observing the distribution of red and blue points helps understand the contribution and direction of each feature towards the model output.

### 2.6 Statistical analysis

Patients were categorized into two groups based on their 28-day mortality status. Continuous variables were summarized using means and standard deviations and compared using *t*-tests (or Wilcoxon rank-sum tests). Categorical variables were presented as percentages of the total and compared using chi-square tests (or Fisher's exact tests). A *P*-value < 0.05 was considered statistically significant. Statistical analyses were conducted using R version 4.2.3 and Python version 3.11.4.

## 3 Results

### 3.1 Baseline characteristics

**Figure 1:**
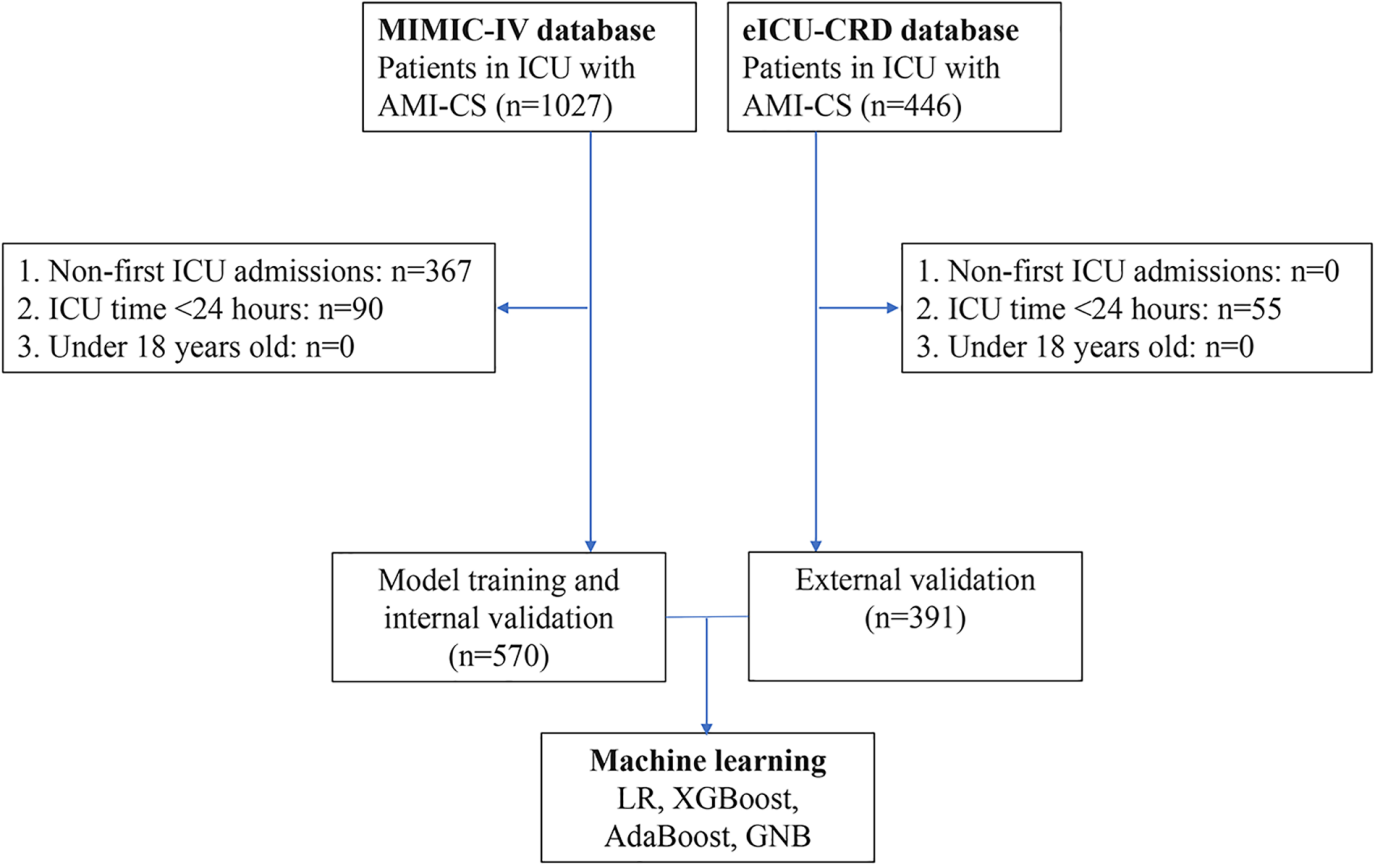
Patient selection flowchart.

According to the inclusion and exclusion criteria, a total of 961 patients with AMI-CS were enrolled in this study, including 570 in the MIMIC-IV database and 391 in the eICU-CRD database. The screening process is illustrated in Figure 1. In the MIMIC-IV database, 215 cases of AMI-CS patients died within 28 days (mortality rate: 37.7%), compared to 102 cases in the eICU-CRD (mortality rate: 26.1%). Differences in baseline characteristics are summarized in Table 1. In both the MIMIC-IV and eICU-CRD databases, patients who died had higher levels of age, blood urea nitrogen (BUN), prothrombin time (PT), creatinine (Cr), international normalized ratio (INR), respiratory rate (R), and APACHE III score, as well as lower levels of serum albumin (ALB) and pH (*p* < 0.05). Furthermore, differences were observed in the use of angiotensin-converting enzyme inhibitors/angiotensin receptor blockers (aceiorarb), beta-blockers, spironolactone, epinephrine, and ventilation (*p* < 0.05) between the survival and mortality groups. However, there were no significant differences in comorbidities such as hypertension, diabetes, and chronic obstructive pulmonary disease (COPD) between the two groups.

**Table 1:** The baseline characteristics of the MIMIC-IV and eICU-CRD databases, categorized by survival and death groups.

	MIMIC-IV			eICU-CRD		
	Survival (*N* = 355)	Death (*N* = 215)	*p*	Survival (*N* = 289)	Death (*N* = 102)	*p*
Age	69.9 (13.1)	75.2 (12.1)	<0.001	66.8 (13.4)	73.8 (10.6)	<0.001
Gender			0.177			1.000
Female	132 (37.2%)	93 (43.3%)		108 (37.4%)	38 (37.3%)	
Male	223 (62.8%)	122 (56.7%)		181 (62.6%)	64 (62.7%)	
BMI	28.7 (5.75)	28.4 (6.81)	0.646	29.0 (6.87)	27.6 (6.77)	0.082
WBC	15.0 (8.53)	14.9 (7.36)	0.872	14.8 (12.0)	17.4 (12.1)	0.065
RBC	3.93 (0.84)	3.64 (0.80)	<0.001	3.99 (0.79)	3.92 (0.76)	0.436
PLT	234 (101)	225 (105)	0.317	209 (83.2)	209 (100)	0.987
Hb	11.7 (2.44)	10.8 (2.26)	<0.001	12.0 (2.28)	11.8 (2.29)	0.633
ALB	3.21 (0.47)	3.04 (0.54)	<0.001	2.97 (0.51)	2.78 (0.58)	0.003
Na	137 (4.92)	138 (5.70)	0.341	137 (4.05)	138 (7.21)	0.578
K	4.42 (0.80)	4.51 (0.90)	0.222	4.17 (0.72)	4.30 (0.85)	0.157
Cl	103 (6.25)	102 (6.66)	0.104	104 (5.66)	104 (8.13)	0.601
Ca	8.30 (0.75)	8.32 (0.92)	0.797	8.21 (0.80)	8.17 (0.79)	0.629
GLU	190 (99.7)	221 (136)	0.004	183 (96.4)	199 (107)	0.175
TBIL	0.85 (0.82)	1.06 (1.12)	0.020	0.96 (0.97)	0.92 (0.59)	0.580
ALT	187 (514)	339 (823)	0.015	210 (956)	312 (619)	0.218
AST	357 (903)	574 (1,368)	0.039	406 (1,350)	573 (1,292)	0.269
BUN	31.0 (20.0)	44.4 (27.8)	<0.001	27.6 (17.1)	38.1 (22.0)	<0.001
Cr	1.59 (1.11)	2.22 (1.71)	<0.001	1.61 (1.57)	2.16 (1.54)	0.002
PH	7.34 (0.10)	7.31 (0.12)	0.002	7.35 (0.09)	7.31 (0.13)	0.004
PO2	122 (101)	127 (105)	0.623	128 (84.2)	143 (105)	0.189
PCO2	40.8 (9.49)	41.1 (11.5)	0.685	40.3 (9.79)	40.2 (15.2)	0.964
PT	16.5 (9.54)	19.6 (11.2)	0.001	16.0 (6.36)	20.9 (12.8)	<0.001
INR	1.52 (1.04)	1.81 (1.05)	0.002	1.42 (0.67)	1.89 (1.50)	0.003
PTT	58.6 (38.4)	65.6 (43.4)	0.053	46.7 (26.1)	52.6 (32.8)	0.099
T	36.7 (0.71)	35.9 (4.79)	0.015	36.6 (0.78)	36.4 (1.18)	0.149
HR	90.1 (20.0)	90.4 (20.7)	0.860	90.0 (19.6)	93.8 (20.4)	0.103
R	19.9 (6.00)	21.4 (7.07)	0.011	19.8 (5.80)	23.0 (7.83)	<0.001
SBP	113 (20.7)	112 (22.0)	0.593	111 (23.4)	111 (24.5)	0.941
DBP	67.0 (18.1)	64.6 (17.7)	0.118	66.0 (17.1)	63.9 (16.6)	0.291
MBP	78.7 (17.4)	76.5 (17.0)	0.142	81.0 (17.4)	79.5 (17.8)	0.448
SpO2	96.0 (4.83)	95.0 (6.77)	0.070	96.6 (5.45)	95.7 (8.14)	0.325
Hypertension			0.070			0.676
0	98 (27.6%)	44 (20.5%)		245 (84.8%)	84 (82.4%)	
1	257 (72.4%)	171 (79.5%)		44 (15.2%)	18 (17.6%)	
diabetes			0.068			0.528
0	222 (62.5%)	117 (54.4%)		278 (96.2%)	100 (98.0%)	
1	133 (37.5%)	98 (45.6%)		11 (3.81%)	2 (1.96%)	
Hyperlipidemia			0.943			0.070
0	166 (46.8%)	102 (47.4%)		251 (86.9%)	96 (94.1%)	
1	189 (53.2%)	113 (52.6%)		38 (13.1%)	6 (5.88%)	
COPD			0.407			0.949
0	320 (90.1%)	199 (92.6%)		266 (92.0%)	93 (91.2%)	
1	35 (9.86%)	16 (7.44%)		23 (7.96%)	9 (8.82%)	
Pneumonia			0.228			0.121
0	276 (77.7%)	177 (82.3%)		250 (86.5%)	81 (79.4%)	
1	79 (22.3%)	38 (17.7%)		39 (13.5%)	21 (20.6%)	
CKD			<0.001			0.141
0	258 (72.7%)	121 (56.3%)		254 (87.9%)	83 (81.4%)	
1	97 (27.3%)	94 (43.7%)		35 (12.1%)	19 (18.6%)	
AF			0.042			0.227
0	209 (58.9%)	107 (49.8%)		231 (79.9%)	75 (73.5%)	
1	146 (41.1%)	108 (50.2%)		58 (20.1%)	27 (26.5%)	
CAG			0.011			0.806
0	230 (64.8%)	162 (75.3%)		184 (63.7%)	67 (65.7%)	
1	125 (35.2%)	53 (24.7%)		105 (36.3%)	35 (34.3%)	
PCIorPTCA			0.076			0.375
0	274 (77.2%)	180 (83.7%)		224 (77.5%)	84 (82.4%)	
1	81 (22.8%)	35 (16.3%)		65 (22.5%)	18 (17.6%)	
IABP			1.000			0.455
0	316 (89.0%)	191 (88.8%)		195 (67.5%)	64 (62.7%)	
1	39 (11.0%)	24 (11.2%)		94 (32.5%)	38 (37.3%)	
Aceiorarb			<0.001			<0.001
0	154 (43.4%)	192 (89.3%)		242 (83.7%)	101 (99.0%)	
1	201 (56.6%)	23 (10.7%)		47 (16.3%)	1 (0.98%)	
Betablockers			<0.001			0.046
0	37 (10.4%)	106 (49.3%)		182 (63.0%)	76 (74.5%)	
1	318 (89.6%)	109 (50.7%)		107 (37.0%)	26 (25.5%)	
Furosemide			0.046			0.001
0	323 (91.0%)	206 (95.8%)		149 (51.6%)	72 (70.6%)	
1	32 (9.01%)	9 (4.19%)		140 (48.4%)	30 (29.4%)	
Spironolactone			0.006			0.025
0	44 (12.4%)	46 (21.4%)		276 (95.5%)	102 (100%)	
1	311 (87.6%)	169 (78.6%)		13 (4.50%)	0 (0.00%)	
Dobutamine			0.201			0.083
0	257 (72.4%)	144 (67.0%)		240 (83.0%)	76 (74.5%)	
1	98 (27.6%)	71 (33.0%)		49 (17.0%)	26 (25.5%)	
Dopamine			<0.001			0.116
0	289 (81.4%)	144 (67.0%)		230 (79.6%)	89 (87.3%)	
1	66 (18.6%)	71 (33.0%)		59 (20.4%)	13 (12.7%)	
Epinephrine			0.015			0.007
0	294 (82.8%)	159 (74.0%)		261 (90.3%)	81 (79.4%)	
1	61 (17.2%)	56 (26.0%)		28 (9.69%)	21 (20.6%)	
Milrinone			<0.001			0.067
0	166 (46.8%)	46 (21.4%)		262 (90.7%)	85 (83.3%)	
1	189 (53.2%)	169 (78.6%)		27 (9.34%)	17 (16.7%)	
Norepinephrine			0.271			0.001
0	253 (71.3%)	143 (66.5%)		179 (61.9%)	43 (42.2%)	
1	102 (28.7%)	72 (33.5%)		110(38.1%)	59(57.8%)	
Phenylephrine			0.789			0.002
0	308(86.8%)	189(87.9%)		248(85.8%)	73(71.6%)	
1	47(13.2%)	26(12.1%)		41(14.2%)	29(28.4%)	
Ventilation			0.002			<0.001
0	20(5.63%)	29(13.5%)		144(49.8%)	28(27.5%)	
1	335(94.4%)	186(86.5%)		145(50.2%)	74(72.5%)	

BMI, body mass index; WBC, white blood cell count; PLT, platelet count; Hb, hemoglobin; ALB, albumin; Na, sodium; K, potassium; Ca, calcium; GLU, glucose; TBIL, total bilirubin; ALT, alanine aminotransferase; BUN, blood urea nitrogen; Cr, creatinine; PO2, partial pressure of oxygen; PCO2, partial pressure of carbon dioxide; PT, prothrombin time; PTT, partial thromboplastin time; T, temperature; HR, heart rate; R, respiratory rate; SBP, systolic blood pressure; SpO2, oxygen saturation; COPD, chronic obstructive pulmonary disease; CKD, chronic kidney disease; AF, atrial fibrillation; PCIorPTCA, percutaneous coronary intervention or percutaneous transluminal coronary angioplasty; IABP, intra-aortic balloon pump; aceiorarb, angiotensin-converting enzyme inhibitors or angiotensin II receptor blockers; Betablocker, beta-blockers.

We removed features with a missing rate exceeding 30%, as shown in the Supplementary Figure S1. Additionally, we illustrated the correlation heat map for features with correlations exceeding 0.5, along with the results of features with VIF exceeding 5, as depicted in the Supplementary Figure S2 and Supplementary Figure S3. For features exhibiting high correlation and VIF, we conducted screening before model construction and excluded the following features: red blood Cell count (RBC), coronary angiography (CAG), aspartate aminotransferase (AST), mean blood pressure (MBP), diastolic blood pressure (DBP), international normalized ratio (INR).

### 3.2 Feature selection

**Figure 2:**
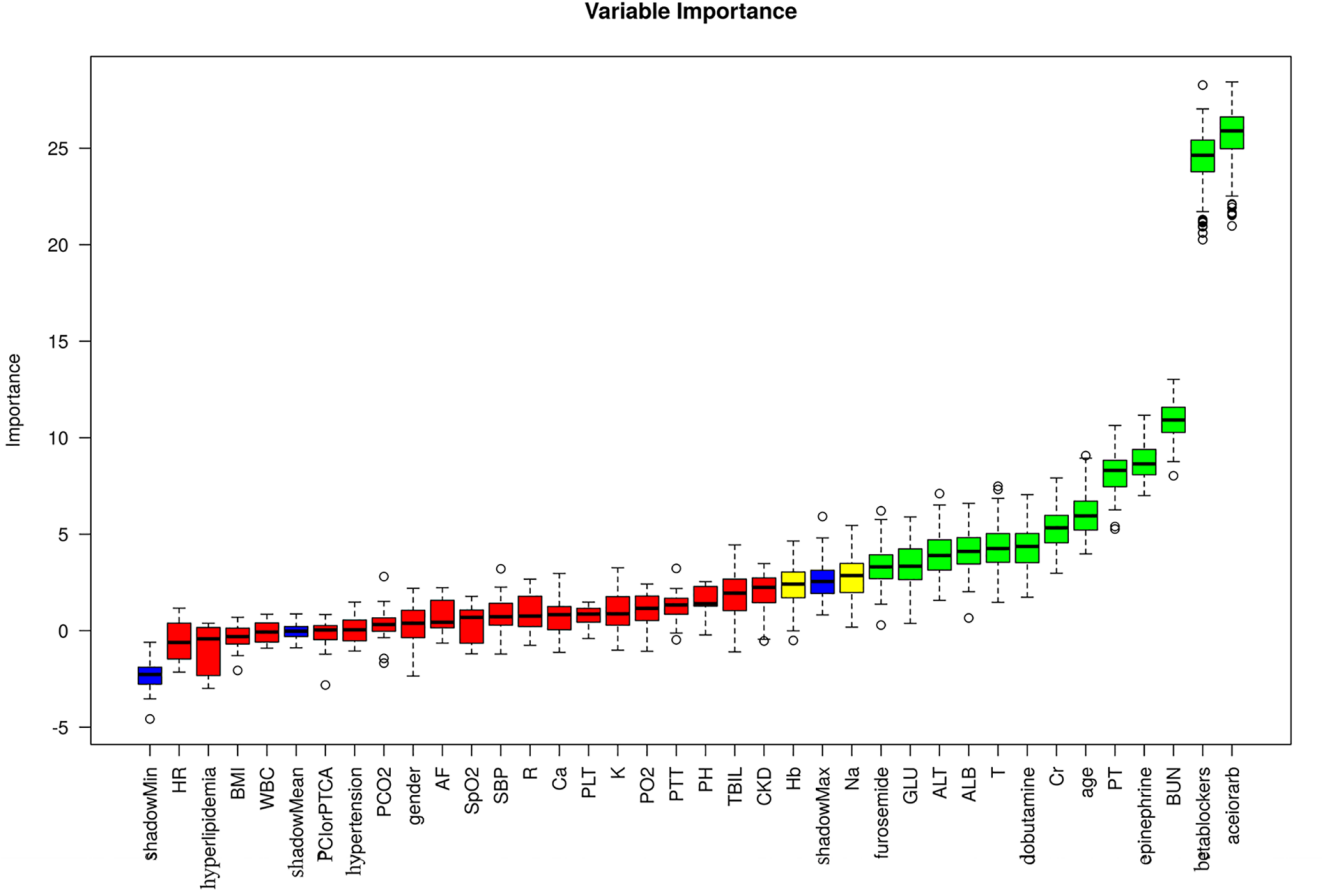
Feature selection analyzed by boruta algorithm.

We employed the Boruta algorithm for feature selection and generated a plot, shown in Figure 2. Boruta assesses feature importance by creating shadow features (derived from shuffling original feature values) and training them alongside original features in a random forest. In the plot, green denotes important features, enhancing model prediction and thus included. Red represents unimportant features excluded from the model, while yellow signifies uncertain importance, necessitating further investigation. Blue indicates shadow features used for comparison but not in model training. Through Boruta selection, we identified 13 features for inclusion: angiotensin-converting enzyme inhibitors or angiotensin II receptor blockers (aceiorarb), beta blockers (betablockers), furosemide, dobutamine, norepinephrine, age, albumin (ALB), glucose (GLU), alanine aminotransferase (ALT), blood urea nitrogen (BUN), creatinine (Cr), prothrombin time (PT), and temperature (T), along with two potential features: hemoglobin (Hb) and sodium (Na).

We utilized the XGBoost method to rank the importance of features, as depicted in Supplementary Figure S4. The top 10 variables, ranked from highest to lowest importance, are: “aceiorarb”, “betablockers”, “PT”, “age”, “BUN”, “GLU”, “T”, “Na”, “dobutamine”, and “Hb”.

### 3.3 Model construction

**Figure 3:**
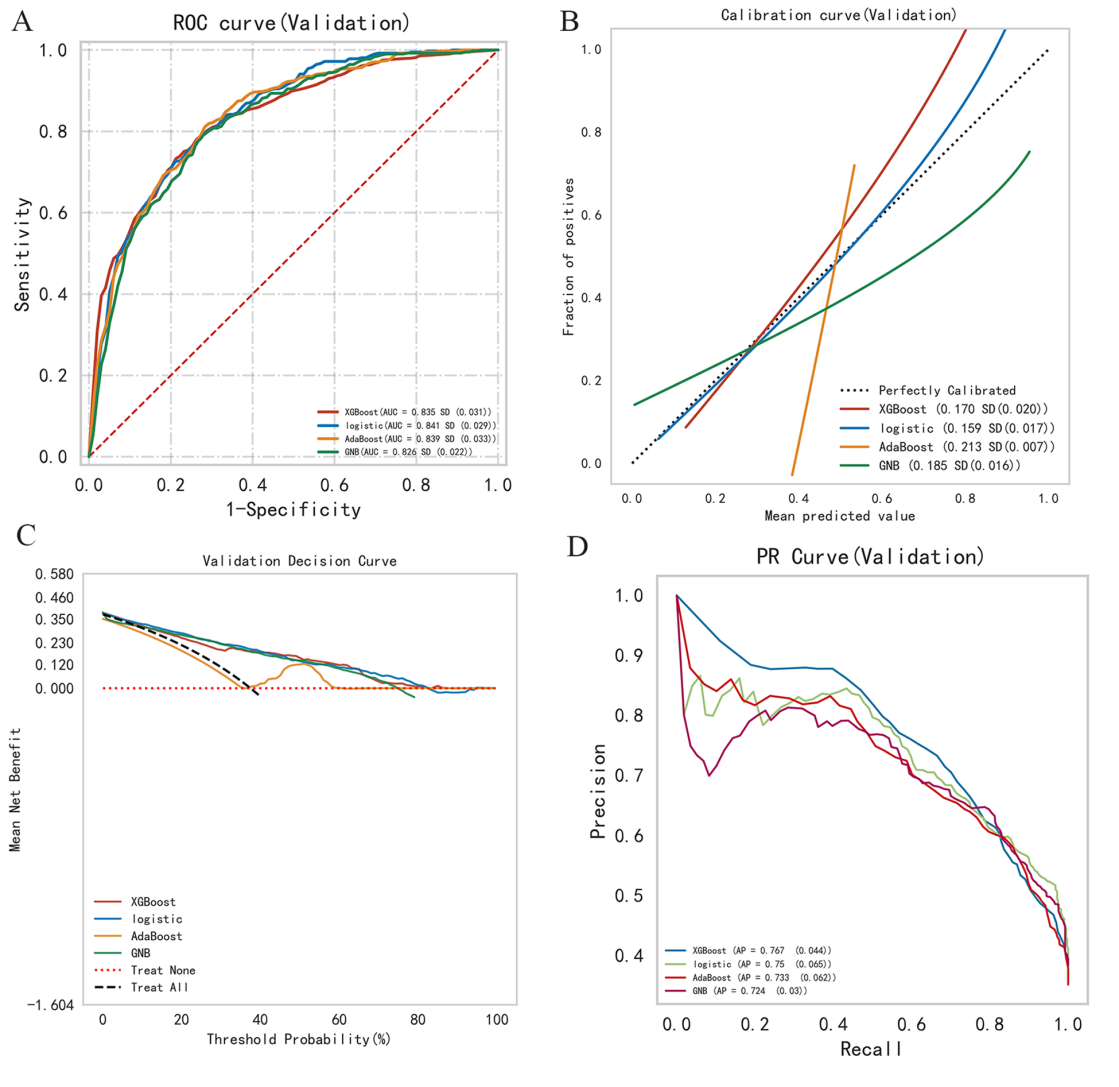
Summary plot of machine learning performance evaluation. **(A)** ROC curve, **(B)** calibration plot, **(C)** DCA curve, **(D)** PR curve.

This study employed four binary classification machine learning algorithms: Logistic Regression (LR), eXtreme Gradient Boosting (XGBoost), Adaptive Boosting (AdaBoost), and Gaussian Naive Bayes (GNB). Utilizing the MIMIC database, we employed a 10-fold cross-validation technique to divide the dataset into training and validation subsets, and evaluated the model performance on the validation subsets. Figure 3A and Table 2 describe the performance of these predictive models, with results indicating that the LR model exhibits better discriminative ability, achieving an AUC of 0.841 in the test queue, compared to other ML models (AUC: XGBoost = 0.835; AdaBoost = 0.839, GNB = 0.826). Furthermore, LR's calibration curve closely approximated the ideal line (Figure 3B), exhibiting the lowest Brier score, indicative of superior calibration. Decision Curve Analysis (DCA) illustrated in Figure 3C revealed LR's highest net benefit within the 0%–80% threshold range. As shown in the PR curve (Figure 3D), the LR model maintains high precision while capturing more positive samples, demonstrating superior performance. Hence, LR was selected for model development, incorporating five predictive variables: betablockers, aceiorarb, PT, age, and BUN. Model parameter optimization through hyperparameter tuning and grid search yielded the following settings: tol (convergence criterion) = 0.0001, penalty (regularization type) = l2, max_iter (maximum number of iterations) = 100, and C (regularization factor) = 10.0.

**Table 2:** Model performance compariso: AUC, accuracy, sensitivity, specificity; PPV, NPV, F1 score, brier score.

Models	AUC	Accuracy	Sensitivity	Specificity	PPV	NPV	F1 Score	Brierscore
Validation set								
XGBoost	0.835	0.767	0.769	0.787	0.687	0.821	0.725	0.170
LR	0.841	0.750	0.799	0.760	0.664	0.816	0.722	0.159
AdaBoost	0.839	0.744	0.804	0.765	0.603	0.864	0.686	0.213
GNB	0.826	0.748	0.805	0.757	0.641	0.842	0.711	0.185
External validation								
LR	0.755	0.630	0.827	0.623	0.580	0.855	0.681	0.229

LR, logistic regression; XGBoost, eXtreme gradient boosting; AdaBoost, adaptive boosting; GNB, gaussian naive bayes; AUC, area under the curve; PPV, positive predictive value; NPV, negative predictive value.

### 3.4 Model interpretation

**Figure 4:**
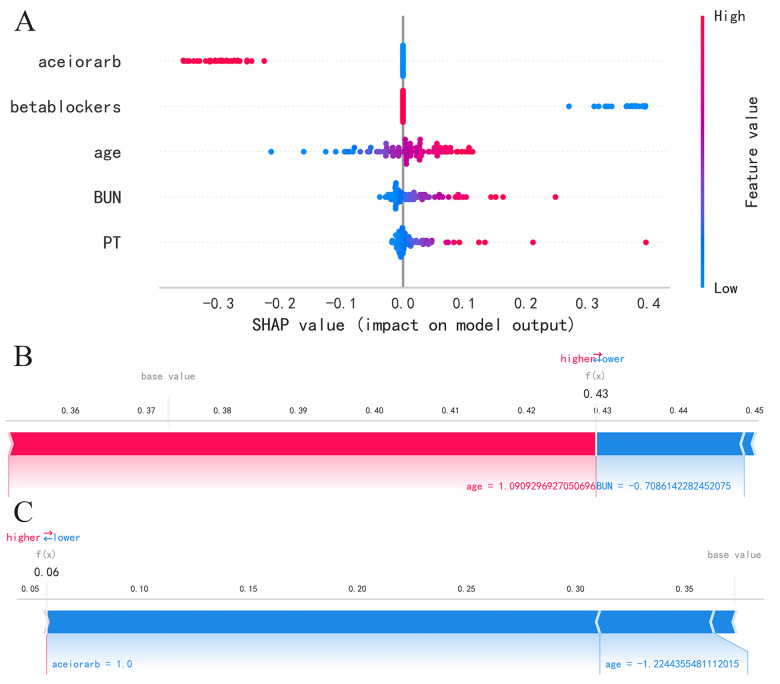
SHAP summary plot and SHAP force plot, **(A)** SHAP summary plot, **(B)** SHAP force plots for patient 1, **(C)** SHAP force plots for patient 2.

We utilize the SHAP method to interpret the model results. For global interpretation, the absolute Shapley values are averaged across all instances in the data, yielding importance values for each feature. The results are visualized as SHAP summary plots, where the *Y*-axis represents the features, and the *X*-axis indicates the magnitude of the feature's impact on the outcome. Each point represents a sample, with red points indicating high-risk values and blue points indicating low-risk values. As shown in SHAP summary plot (Figure 4A), the feature importance from top to bottom is: aceiorarb, betablockers, age, BUN, PT. Patients using aceiorarb (red points) have a reduced risk of death, while patients not using betablockers (blue points) have an increased risk of death. Similarly, higher values of age, BUN, and PT are associated with higher risk of death.

For local interpretation, each observation has its own set of Shapley values, which can be utilized to explain the contribution of each sample and feature to the prediction. The results are visualized as SHAP force plots, where each Shapley value is represented as an arrow that can either push the prediction up (positive value) or down (negative value). Due to the standardization of numerical variables, the age, BUN, and PT values in the plot do not represent the actual values of individuals. As depicted in Figure 4B for patient 1, as age increases, the predicted mortality risk rises, while a decrease in BUN value corresponds to a decrease in predicted mortality risk. As depicted in Figure 4C for patient 2, a decrease in age and the use of ACEI or ARB drugs are associated with a reduction in predicted mortality risk.

### 3.5 Model deployment and external validation

**Figure 5:**
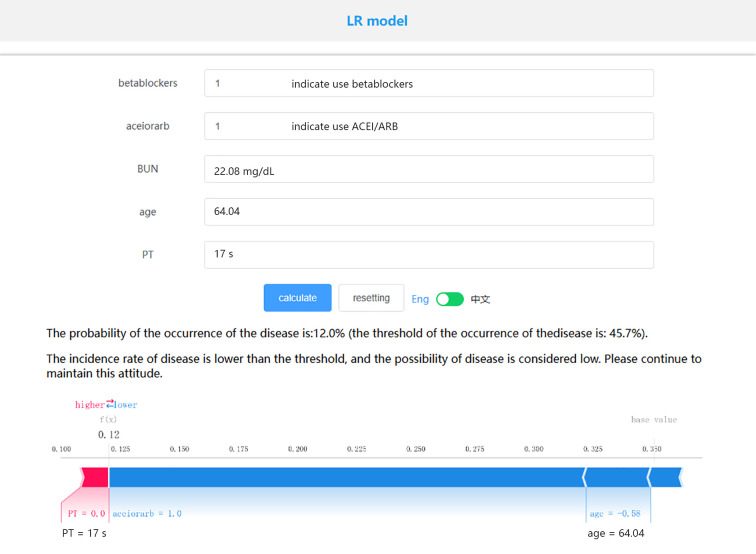
An online prediction model based on LR algorithm.

Based on the LR model, which exhibited the best performance, we established an online prediction model. As shown in Figure 5, we present the survival prediction for actual patients using the online website. The patient is 64 years old, with PT of 17s, BUN of 22.08 mg/dl, has used ACEI/ARB, and has used beta-blockers. The probability of the occurrence of the disease is: 12.0% (the threshold of the occurrence of the disease is: 45.7%). To facilitate understanding, we randomly selected four survivors and four deceased patients from the MIMIC-IV and eICU databases in the past. We had used the online prediction model to estimate their mortality risk. According to the LR model, when the predicted mortality risk was less than 50%, the patient was inferred to have survived beyond 28 days, and when it was greater than 50%, the patient was inferred to have died within 28 days. The final results, which were presented in Supplementary Table S3, demonstrated a 100% accuracy in predicting the outcomes for these eight patients. We extracted 391 cases of AMI-CS patients from the eICU-CRD database as an external validation dataset. The data characteristics are outlined in Table 2. The 28-day in-hospital mortality rate in the eICU-CRD database was 26.1%, lower than that in the MIMIC database. Prior to external validation, we processed the eICU-CRD data using the same methods as the MIMIC data. Due to the substantial imbalance between survival and death cases, we applied Synthetic Minority Over-sampling Technique (SMOTE) to balance the dataset. The results of external validation revealed an AUC of 0.755 and a Brier score of 0.229. Other metrics for external validation, such as accuracy, sensitivity, etc., are available in Table 2. Additionally, an analysis of commonly used severity scores using the MIMIC-IV dataset revealed model AUC values, including SOFA (AUC = 0.620), APSIII (AUC = 0.710), SAPSII (AUC = 0.660), and OASIS (AUC = 0.640).

## 4 Discussion

In this paper, we introduce for the first time an interpretable machine learning algorithm for predicting in-hospital mortality in patients with AMI-CS. This novel model outperforms traditional prediction tools by effectively managing complex datasets and identifying intricate nonlinear relationships. It demonstrates superior accuracy in differentiating between survival and death outcomes of patients, and shows commendable performance in terms of calibration and clinical utility. The high interpretability of the model facilitates ease of understanding and application of its results. Moreover, the model utilizes a minimal number of easily accessible predictive variables, which enhances its practicality in clinical settings.

Several retrospective studies have utilized critical illness scoring systems, including APACHE II, APACHE III, SAPS II, and SOFA, to predict the in-hospital mortality risk of patients with AMI-CS (13, 14). However, the discriminative ability of these models only falls within an AUC range of 0.67 to 0.79. Additionally, the models have included a sample size of fewer than 100 individuals and lack validation cohorts. The IABP-SHOCK II scoring system, derived from multicenter, randomized, controlled trial data, is designed to assess the short-term mortality risk in patients with AMI-CS (16). It effectively categorizes patients into low, moderate, and high-risk groups. However, the model is based on a limited cohort study (*n* = 480) and has only been validated in two small sample sizes of 137 and 98 patients, respectively. Consequently, this model offers a valuable tool for physicians to more precisely evaluate the risk profiles of AMI-CS patients and implement timely interventions to improve their prognoses. CardShock scoring model exhibits good discriminative ability in predicting short-term mortality risk in CS (AUC = 0.85). However, in external cohorts, the AUC of the CardShock scoring model drops to 0.71, suggesting potential overfitting (26). Our model outperforms common critical illness scoring systems. Additionally, with a larger cohort of AMI-CS patients, it demonstrates strong performance in both training and validation sets and shows good generalization ability in external validation.

Our study shows that the use of ACEI/ARB and beta-blockers are significant prognostic factors for mortality in AMI-CS patients. However, these results should be interpreted with caution, as they do not imply a causal relationship between the use of these medications and the prognosis of AMI-CS patients. It is likely that patients who were able to use these medications were in better overall condition, which could explain their better outcomes. These medications are identified as the two most important predictive factors. ACEI/ARB can dilate blood vessels by inhibiting the renin-angiotensin system, reducing cardiac load, and improving myocardial remodeling. Potential mechanisms may involve early inhibition of neurohormonal activation and reduction in infarct size, as well as an increase in regional wall motion and collateral coronary flow (27, 28). Beta-blockers reduce myocardial oxygen demand by attenuating sympathetic nervous system activity, leading to improved cardiac function and prognosis by suppressing ventricular arrhythmias (29). A retrospective study of 4,478 AMI patients with SBP < 100 mmHg showed that early use of ACEI/ARB significantly reduced MACE occurrence compared to non-users (1.67% vs. 3.66%) (30). The timing of initiating ACEIs/ARBs and beta-blockers in AMI patients with low blood pressure is controversial in clinical practice due to their hypotensive effects. The expert consensus recommends that in coronary heart disease patients, if hypotension (systolic blood pressure <90mmHg) occurs during ACEI treatment and the patient is asymptomatic, ACEI should be continued (31). Beta-blockers are recommended as one of the first-line medications for improving mortality rates in patients with chronic heart failure (32, 33). As is well known, beta-blockers are contraindicated in overt heart failure or low-output states due to their negative inotropic effects. Research has indicated that reducing the dosage or discontinuing beta-blockers in heart failure patients previously treated with them may lead to adverse outcomes (34). However, a meta-analysis found that although beta-blockers reduce recurrent myocardial infarction, they increase the risk of cardiogenic shock, without providing a mortality benefit in treating AMI (35). Similarly, a large randomized controlled trial indicated that early use of beta-blockers in acute myocardial infarction reduces the risk of recurrent infarction and ventricular fibrillation but increases the risk of cardiogenic shock (36). Hence, the general prudent approach is to initiate beta-blocker therapy in the hospital setting only after achieving hemodynamic stability following myocardial infarction.

Our study findings indicate that older age is associated with a higher predicted risk of mortality, consistent with the CardShock risk score (26). As age increases, there's a decline in cardiac and organ function. Elderly individuals are more likely to have multiple chronic diseases, reducing their ability to cope with and recover from cardiogenic shock, increasing mortality risk. Cinar et al. found that age remains an important risk factor for mortality in AMI-CS patients (37). Ming-Lung et al.'s study finds a significant positive correlation between age and risk, showing that STEMI patients aged 85 and above have a 3.42 times higher short-term risk compared to those under 55 years old (38). The study suggests that higher PT levels may be associated with poorer risk prediction. Similarly, in mortality prediction models for acute myocardial infarction, PT was observed as one of the top 8 predictors for predicting death in AMI patients (39). Prolonged PT in these patients may indicate a higher risk of mortality as it reflects disruptions in the coagulation system, possibly leading to severe complications. Additionally, it may serve as a marker of systemic illness severity and multi-organ dysfunction, correlating with elevated mortality risk.

Our study indicates that higher BUN levels are associated with an increased risk of mortality in AMI-CS patients, while creatinine clearance rate is not a significant predictor. Yuan et al.'s study of 218 AMI-CS patients showed that higher admission BUN levels, particularly those exceeding 8.95 mmol/L, were independently associated with a greater risk of 30-day mortality (40). Some research suggests that kidney function plays a crucial role in predicting the prognosis of AMI-CS patients. In a subgroup analysis of the IABP-SHOCK II trial, serum creatinine levels were found to be a significant independent predictor of one-year mortality (41). In the TRIUMPH multicenter trial, higher creatinine clearance rates were associated with lower 30-day mortality in univariate analysis (odds ratio 0.77) (42). In heart failure patients, BUN has been observed to have the highest predictive efficacy for predicting 30-day mortality among BUN, creatinine, BUN-to-creatinine ratio, and GFR (43–45). Creatinine is filtered in the renal glomeruli and not reabsorbed, while urea is reabsorbed in the renal tubules. A decrease in urine flow in AMI-CS patients can lead to increased urea reabsorption, resulting in higher BUN levels, which may be a more sensitive indicator.

This study has several limitations. Firstly, being a retrospective study, despite conducting internal and external validation using two databases, it's challenging to completely avoid selection bias. Further multicenter and large-scale clinical studies are warranted. Secondly, the clinical information collected is limited. Future research should delve into and integrate imaging modalities such as echocardiography, lung CT, and coronary imaging results to enhance prediction accuracy. Lastly, our data originates from ICU patients, potentially differing from those in other cardiovascular units. Therefore, additional validation is necessary to ascertain its applicability to other cardiovascular units.

## 5 Conclusion

This article uses machine learning algorithms to construct models that can accurately predict the in-hospital mortality risk of patients with AMI-CS in the ICU. The LR algorithm demonstrates the best predictive performance, clear result interpretation, and the predictive variables are easily accessible, offering valuable guidance for clinical practice.

## Data Availability

The original contributions presented in the study are included in the article/Supplementary Material, further inquiries can be directed to the corresponding author.
