# Supplementary material for: Machine learning-based prediction of mortality in acute myocardial infarction with cardiogenic shock
Source: Front Cardiovasc Med. 2024 Oct 14;11:1402503. doi: 10.3389/fcvm.2024.1402503 (PMC11513311; doi:10.3389/fcvm.2024.1402503)
Supplement: Supplementary file 1 [file Table1.pdf]

Supplementary Table 1: Survival Prediction Using the LR Model on MIMIC-IV and eICU Datasets

age	age input	BUN	BUN input	PT	PT input	aceiorarb	betablockers	death	Predicting Mortality Risk
MIMIC									
89	1.50	139	4.29	74.6	5.76	0	0	1	97.6%
84	1.08	112	3.17	27.9	1.09	0	1	1	75.8%
91	1.67	29	-0.29	13.7	-0.33	0	1	0	46.5%
57	-1.17	19	-0.71	14.5	-0.25	1	1	0	9.5%
eICU									
75	0.33	57	0.88	14.4	-0.26	0	0	1	73.2%
84	1.08	43	0.29	13.1	-0.39	0	0	1	59.1%
70	-0.08	16	-0.83	10.9	-0.61	0	1	0	29.7%
64	-0.58	22	-0.58	17	0	1	1	0	12.0%

Explanation: The online prediction model website is
https://www.xsmartanalysis.com/model/list/predict/model/html?mid=13163&symbol=41mZhnEL7Yr100695823.

Model input description:

parameter description

betablockers: Input 0 or 1, where 0 indicates non-usage of betablockers medication, and 1 indicates usage.

aceiorarb: Input 0 or 1, where 0 indicates non-usage of ACEI/ARB medication, and 1 indicates usage.

BUN: Input y, y = (x - 36) / 24; (x represents the actual BUN)

age: Input y, y = (x - 71) / 12; (x represents the actual age)

PT: Input y, y = (x - 17) / 10; (x represents the actual PT)
