# Supplementary figures and images for: Machine learning-based prediction of mortality in acute myocardial infarction with cardiogenic shock
Source: Front Cardiovasc Med. 2024 Oct 14;11:1402503. doi: 10.3389/fcvm.2024.1402503 (PMC11513311; doi:10.3389/fcvm.2024.1402503)

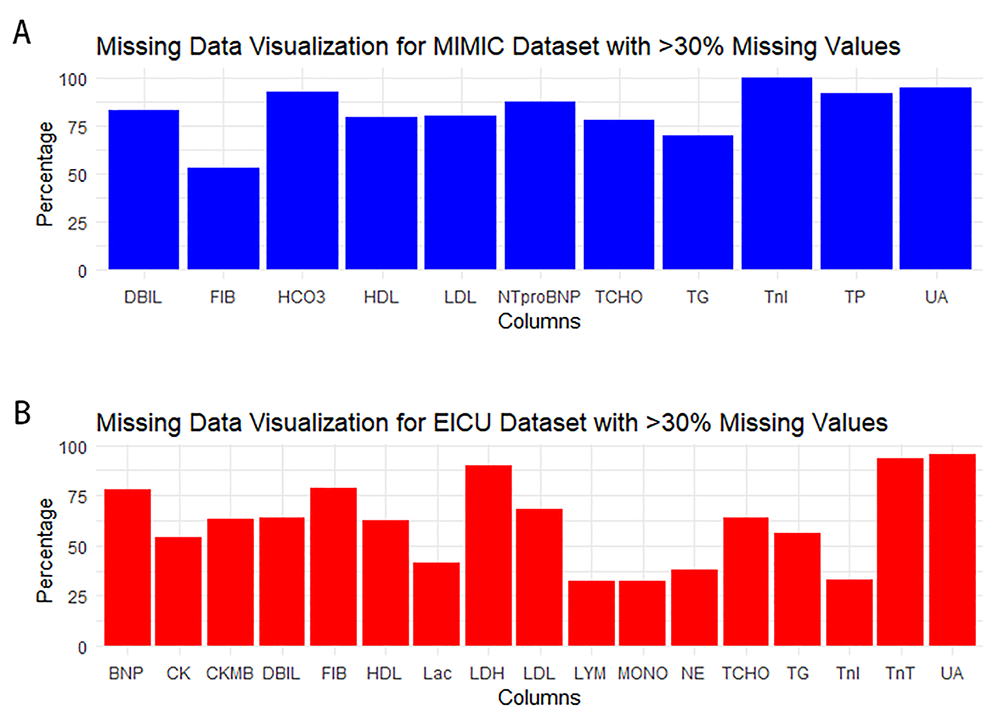

Supplement: Supplementary file 2 [file Image1.tif]

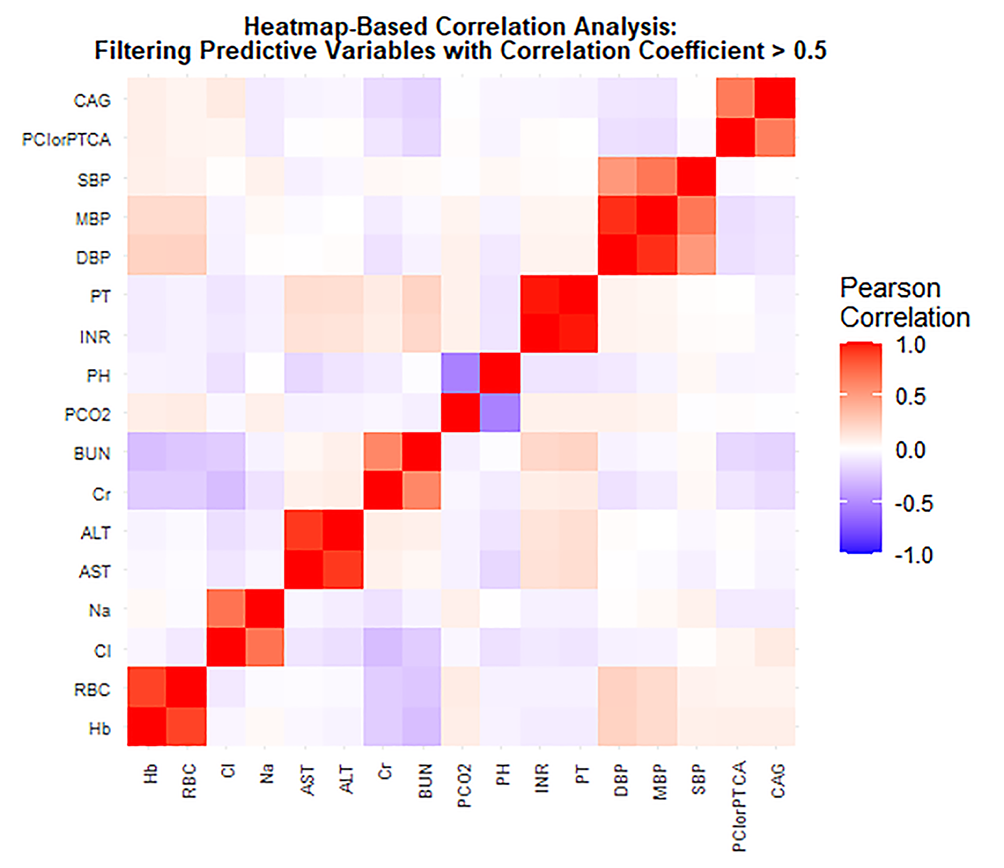

Supplement: Supplementary file 3 [file Image2.tif]

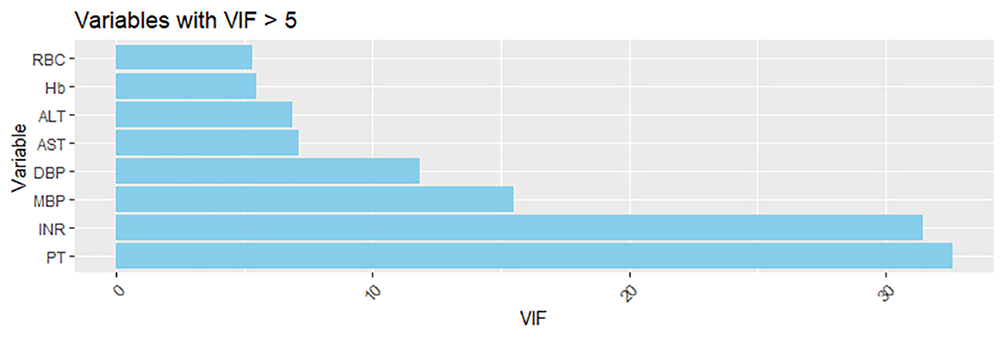

Supplement: Supplementary file 4 [file Image3.tif]

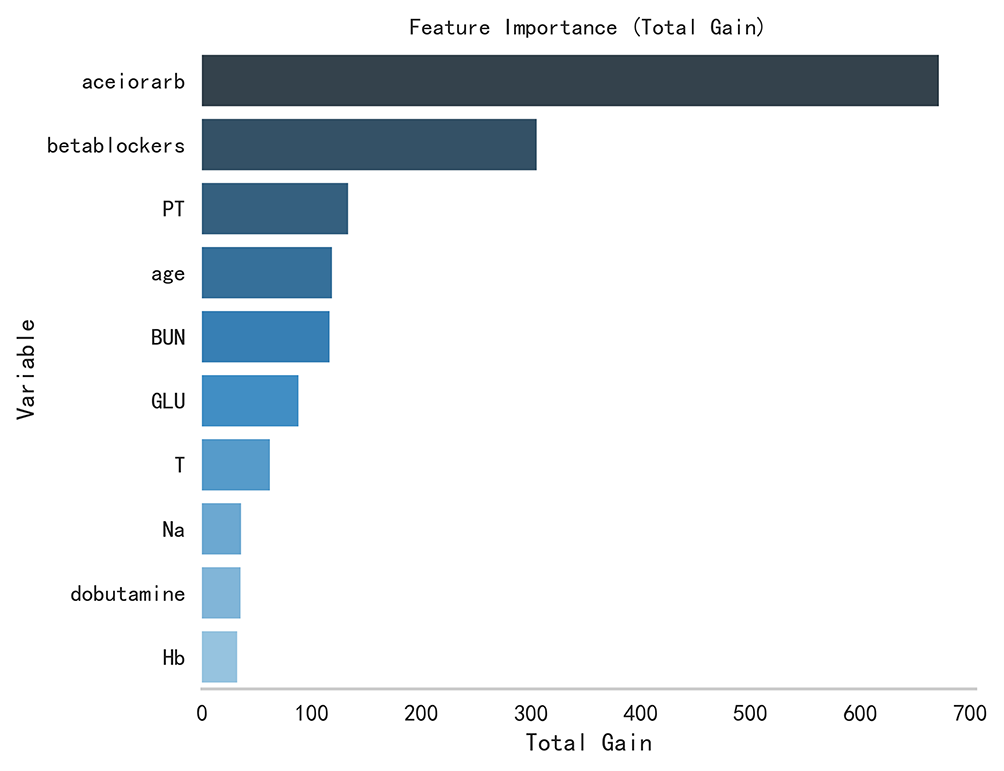

Supplement: Supplementary file 5 [file Image4.tif]
